# Emotion Regulation Questionnaire-Adapted and Individual Differences in Emotion Regulation

**DOI:** 10.5964/ejop.2755

**Published:** 2021-02-26

**Authors:** Rita Seixas, Anne Pignault, Claude Houssemand

**Affiliations:** a Department of Education and Social Work, Institute for Lifelong Learning and Guidance, University of Luxembourg, Luxembourg; b Laboratory of Psychology & Neurosciences (2LPN, EA7489), Université de Lorraine, Nancy, France; University of Bari Aldo Moro, Bari, Italy

**Keywords:** emotion regulation, expressive enhancement, individual differences, personality

## Abstract

Emotion regulation is a human adaptation process with important implications for daily life. Two specific emotion regulation strategies were the principle areas of study: reappraisal (cognitive change in which individuals adapt their state of mind about a given situation) and expressive suppression (response modulation in which individuals change their emotional response after its initiation). The Emotion Regulation Questionnaire (ERQ), that captures individual tendencies to reappraise and to suppress the expression of emotions, was also developed. Response modulation strategy was analyzed by considering two distinct processes: expressive suppression (down-regulation) and expressive enhancement (up-regulation). This latter modulation process has been less frequently studied by researchers. The present study investigates the psychometrical properties, individual differences and correlates of a French adapted version of the ERQ, which comprises reappraisal and the two response modulation tendencies – expressive suppression and expressive enhancement. Based on the initial ERQ, new items were created and added to the scale. The three-factor structure of the ERQ adapted was confirmed. As expected, emotion regulation is linked to individual differences: the tendency to reappraise has a positive low correlation with age; and men are significantly more disposed to suppress and to enhance than women. Finally, the tendency to suppress the expression of emotions is negatively correlated with extraversion, and the disposition to enhance the expression of emotions is negatively correlated with conscientiousness.

Emotions are central to human life (Cosmides & Tooby, 2000; Frijda, 1987; Keltner & Kring, 1998; Williams et al., 2004, Phelps, 2006) but they can be unhelpful if they occur at inconvenient moments or with an inappropriate intensity level (Gross, 1998; Gross & Thompson, 2007). Emotion regulation is currently a frequently studied research domain, with numerous studies attesting to its importance for human adaptation, and its implications for people’s lives (Gross, 2002, 2015; Gross & John, 2003; Gross & Muñoz, 1995; John & Gross, 2004; Mikolajczak, Tran, Brotheridge, & Gross, 2009; Snyder, Simpson, & Hughes, 2006).

Emotion regulation refers to “the processes by which individuals influence which emotions they have, when they have them and how they experience and express them” (Gross, 1998, p. 275). It can be automatic or effortful, conscious or unconscious, intrinsic (to regulate one’s own emotions) or extrinsic (to regulate others’ emotions) (Gross & Thompson, 2007). According to Gross´s Process Model of Emotion Regulation (Gross, 1998), individuals can use numerous strategies to regulate their emotions. These can be organized according to the moment at which they intervene in the emotion-generative process, regardless of their potential adaptive or maladaptive value (Gross & Thompson, 2007). Five families of emotion regulation strategies can be differentiated: *situation selection*, *situation modification*, *attentional deployment*, *cognitive change*, and *response modulation* (Gross, 1998, 2008; Gross & John, 2003; John & Gross, 2004). The first four families of strategies are considered *antecedent-focused* because they take place before the emotion response tendency is completely activated. The last family of strategies (response-modulation strategies) are considered *response-focused* because they are activated after an emotion has started to manifest itself and a response tendency has already been generated (Gross, 1998; John & Gross, 2004). Gross and colleagues developed a vast body of research on two families of strategy – cognitive change and response modulation – and more specifically on two specific strategies: reappraisal (cognitive change) and expressive suppression (response modulation). Reappraisal is an antecedent-focus strategy, as it occurs before the emotion is installed. It refers to a cognitive process in which individuals change the way they think about a situation in order to change (by increasing – “up-regulation” or by decreasing – “down-regulation”) its emotional significance and impact (Gross, 2008). For example a person noticing that his/her best friend forgot to wish her a happy birthday can create feelings of disappointment or sadness. Reappraising the situation to down-regulate negative emotions would involve finding an alternative explanation for this forgetfulness, such as an excess of work or family-related problems, instead of seeing it as a neglect. Suppression is a response-focused strategy as it occurs after the emotion is installed. It consists of changing the (experiential, behavioral, or physiological) emotional response after it has been initiated (Gross, 2008). An example is the efforts made by a person to hide her anxiety in an oral presentation.

## The Emotion Regulation Questionnaire

With the purpose of capturing individual differences in cognitive reappraisal and expressive suppression, Gross and colleagues developed the Emotion Regulation Questionnaire (ERQ; Gross & John, 2003). The ERQ comprises 10 items capturing individuals’ tendencies (self-perceived frequencies) to reappraise and to suppress the expression of emotions. Respondents are presented with statements regarding the use of reappraisal and suppression. They indicate their agreement or disagreement on a rating scale, ranging from 1 for “*strongly disagree*” to 7 for “*strongly agree*”. In the development of the ERQ items, care was paid to not associate any strategy with affective, social or well-being consequences, as these could influence individuals’ responses. The validity, reliability and factor structure of this questionnaire has been demonstrated (Gross & John, 2003) and replicated consistently (e.g., Brandão, Schulz, Gross, & Matos, 2017; Ireland, Goh, & Marais, 2018; Melka, Lancaster, Bryant, & Rodriguez, 2011; Moore, Zoellner, & Mollenholt, 2008; Peng, Min, & Chen, 2016). Regarding its factor structure, these two strategies have been shown to be statistically independent. This means that they should not be seen as being negative (using one does not imply not using the other), nor as positively dependent (the frequent use of one of them has no influence on the tendency or disposition to use the other; Gross & John, 2003). Research has been interested in studying the consequences of their use, individual differences in their regard and their links to related constructs such as personality.

## Consequences of Reappraisal and Expressive Suppression

The self-perceived use of reappraisal and expressive suppression have distinct affective, cognitive and social consequences:

- *affective consequences*: reappraisal is linked to subjective well-being (Ortner, Briner, & Marjanovic, 2017), lower depression and lower trait anxiety, while expressive suppression is linked to lower well-being and greater negative emotions, feelings of inauthenticity and depression (Aldao, Nolen-Hoeksema, & Schweizer, 2010; Egloff, Schmukle, Burns, & Schwerdtfeger, 2006; Gross & John, 2003; Nezlek, Vansteelandt, Van Mechelen, & Kuppens, 2008);
- *cognitive consequences*: frequent “suppressors” have poorer self-reported and objective memory than those who suppress less frequently, while “reappraisers” have comparable or enhanced memory than “non-reappraisers” (Richards & Gross, 2000);
- *social consequences*: the habitual use of suppression is correlated with lower levels of social connection (social support and closeness), while reappraisal is positively linked to peer-rated social connection, both using cross-sectional (Gross & John, 2003) and longitudinal databases (English, Gross, John, & Srivastava, 2012).

Even though these data suggest that suppressing the expression of emotions has worse outcomes than expressing or reappraising situations, there is also evidence on the adaptive benefits of suppression (Gross & Muñoz, 1995; Keltner, Kring, & Bonanno, 1999) and on the maladaptive effects of reappraisal (Troy, Shallcross, & Mauss, 2013). For example, to inhibit the expression of emotions can lead to better long-term adjustment in adverse contexts (Bonanno & Keltner, 1997). Reappraisal is only related to depression in highly stressful contexts (Troy, Wilhelm, Shallcross, & Mauss, 2010) and its effects can be deleterious if the stress is high but controllable (Troy et al., 2013). Taken together these findings support a functionalist perspective (Nesse, 1990), according to which emotion regulation strategies are not adaptive or maladaptive per se, but their impact varies according to the situational/context variables and the goals of the individual (Gross, 2013; Troy et al., 2013).

## Individual Differences in Reappraisal and Expressive Suppression

Do men and women differ in their self-perceived use of reappraisal and suppression? Does its use vary over the course of a life? These are some of the questions that have merited researchers’ attention. As regards gender differences, studies are generally consistent in demonstrating that men and women differ in their use of emotion regulation strategies (Hess et al., 2000; Nolen-Hoeksema & Aldao, 2011; Tamres, Janicki, & Helgeson, 2002; Zimmermann & Iwanski, 2014). Specific data on the original ERQ indicates that men use expressive suppression significantly more frequently than women, while the use of reappraisal does not seem to differ between genders (Gross & John, 2003). These results on the use of suppression to regulate (by decreasing) an experienced emotion are in line with studies showing that men tend to conceal the expression of emotions more than women (Nolen-Hoeksema & Aldao, 2011; Tamres et al., 2002). However, men and women reappraise with the same frequency, giving support to the view that the use of antecedent and more cognitive-based strategies are not a gender characteristic.

In terms of its development, emotion regulation is conceptualized as changeable in time as individuals develop and learn from life experiences, and perceive the costs and benefits of different emotion regulation strategies (Blanchard-Fields & Coats, 2008; Blanchard-Fields, Stein, & Watson, 2004; John & Gross, 2004; Nolen-Hoeksema & Aldao, 2011). As previously mentioned, reappraisal has been shown to be associated with healthier outcomes than suppression (Gross & John, 2003). Thereby it is expected that the use of reappraisal increases with age, while the use of suppression decreases. Longitudinal and cross-sectional studies using the ERQ support this idea. They report that the use of reappraisal increases from early adulthood (20s) to later adulthood (60s), while the use of suppression decreases (John & Gross, 2004). These age related changes are arguably related to both a change in the contexts faced by younger and older adults, and to a learning process which leads to a higher use of strategies linked to better consequences, to the detriment of strategies linked to more negative consequences (Gross & Thompson, 2007).

## Links between Personality Traits and Reappraisal and Expressive Suppression

Individuals differ in their personality traits, these distinct affective predispositions influence the use of emotion regulation strategies, specifically by facilitating or making it difficult to use reappraisal and expressive suppression (John & Gross, 2004). Even if there is no consensus about the nature of these links (Balzarotti, John, & Gross, 2010; Cabello, Salguero, Fernández-Berrocal, & Gross, 2012; Gresham & Gullone, 2012; John & Gross, 2008; Larsen & Ketelaar, 1991; Ng & Diener, 2009; Páez, Seguel, & Martínez-Sánchez, 2013), some results are recurrent throughout the studies: expressive suppression is negatively related to extraversion, and reappraisal is negatively related to neuroticism. Extraverts actively seek social interactions, use their emotions constructively to change situations (John & Gross, 2004), and are more likely to express emotions than introverts do (Gross & John, 1998). This contrasts with introverts who tend to be more self-conscious and sensitive to rejection cues from others. This would explain a tendency to suppress the expression of emotions in order to avoid potential rejection (Ayduk et al., 2000). The negative correlation between neuroticism and reappraisal is in line with the fact that emotionally unstable individuals tend to experience strong negative emotions and to feel overwhelmed by their feelings (Wang, Shi, & Li, 2009). This characteristic makes it less likely for these individuals to use cognitive strategies like reappraisal, as it requires being capable of adopting alternative and constructive points of view in order to regulate their emotions (Gross & John, 2003).

## Ways of Improving the ERQ

Given its strong psychometrical properties (Brandão et al., 2017; Enebrink, Björnsdotter, & Ghaderi, 2013; Gross & John, 2003; Ioannidis & Siegling, 2015; Ireland et al., 2018; Melka et al., 2011; Peng et al., 2016) and its easy application, the ERQ is used frequently in studies of individual differences in the self-perceived use of reappraisal and expressive suppression (Balzarotti et al., 2010; Cabello et al., 2012; Gross & John, 2003; Moore et al., 2008; Uphill, Lane, & Jones, 2012). However, expressive suppression represents one “side” of response modulation. In fact, individuals can modulate their emotional responses by down-regulating them (using expressive suppression – for example when avoiding laughing at a friends´ joke during a funeral) but also by up-regulating them (using expressive enhancement – for example when a boxer tries to cultivate feelings of anger before entering the ring) (Gross, 2014). These two strategies represent very distinct (opposed) ways of modulating ones´ emotional responses, which present distinct patterns of individual differences and correlates. Less investigation has been undertaken on expressive enhancement, however some studies have investigated this emotion regulation strategy both at the dispositional (self-reported) and ability levels. For example, being *disposed* to regulate positive emotions by amplifying their expression promotes goal attainment in interactions with superiors at work (Wong, Tschan, Messerli, & Semmer, 2013), leads to lower emotional exhaustion and higher professional efficacy (Andela, Truchot, & Borteyrou, 2015), and to greater job satisfaction (Côté & Morgan, 2002), and being *capable* of enhancing the expression of emotions is linked to greater well-being, and higher disposable income and socioeconomic status (Côté, Gyurak, & Levenson, 2010). These studies are motivation for further research.

The ERQ does not include the assessment of expressive enhancement. This scale can nevertheless be adapted easily in order to add a third dimension comprising self-perceived use of expressive enhancement. Being able to use an adapted version of the ERQ is potentially useful for researchers interested in studying response-modulation, both in terms of down-regulation (expressive suppression) and up-regulation (expressive enhancement).

## The Present Study

This study sought to investigate the psychometrical properties, individual differences and correlates of a French adapted version of the ERQ. It comprises reappraisal and two response modulation tendencies – expressive suppression and expressive enhancement. More specifically, it investigates the following hypothesis:

H1 – the ERQ-adapted presents adequate internal consistency both overall and for each of its subscales (comparable to the internal consistency of the original scale: e.g., Christophe, Antoine, Leroy, & Delelis, 2009; Gross & John, 2003);

H2 – the ERQ-adapted reveals a three-factor structure (exploratory factor analysis) with an appropriate fit to the data (exploratory factor and item-response theory analysis: e.g. Brandão et al., 2017; Gross & John, 2003; Ireland et al., 2018; Melka et al., 2011; Peng et al., 2016);

H3 – reappraisal and suppression within age and gender differences replicate previous research: (H3a) men suppress significantly more frequently than women, while no significant differences exist in the disposition to reappraise (H3b). Individuals’ tendency to reappraise increases with age, while the tendency to suppress decreases. Individual differences in expressive enhancement are explored. To our knowledge, no data exists regarding gender differences on the self-perceived use of expressive enhancement to regulate one’s own emotions. However, on the basis of previous evidence pointing out that women are more emotionally expressive than men (who present a more controlled display of emotions; Buck, Miller, & Caul, 1974; Kret & De Gelder, 2012; Kring & Gordon, 1998), this study investigates the hypothesis that women will up-regulate their inner emotions by enhancing their expression more frequently than men (H3c). Regarding age, despite the lack of dispositional (self-reported) data on expressive enhancement strategy, research at the ability level indicates that individuals capable of using this strategy report greater well-being, as well as higher disposable income and socioeconomic status (Côté et al., 2010). The fact that this strategy is linked with positive outcomes, allied to the fact that individuals’ ability to use this strategy is not affected by age (Vieillard, Harm, & Bigand, 2015), make us expect that expressive enhancement disposition will also not be affected by age (H3d).

H4 – the linkage of reappraisal and suppression with personality replicates previous research: expressive suppression is negatively related to extraversion, and reappraisal is negatively related to neuroticism (e.g., Cabello et al., 2012; Gresham & Gullone, 2012; Gross & John, 2003; Páez, Seguel, & Martínez-Sánchez, 2013). Individual differences in expressive enhancement are explored, with us expecting to observe that because this strategy requires a disposition to disclose and experience more intensively one’s own emotions. We explore the hypothesis that extraverts (as they openly express both positive and negative emotions and use them to modify situations) (H4a), and those with low conscientiousness (who are less governed by conscience/reason, and exert less control over emotional experiences), will report a greater use of expressive enhancement (H4b).

## Method

### Participants and Procedure

A sample of 255 adults in paid employment (152 females; 97 males; 6 missing) aged between 17 and 68 years (*M* = 37.71, *SD* = 11.41) participated in this study. The sample was recruited through collaboration with two professional organizations in Luxembourg (one in finance and one in the health-care sector) and through public advertisements. Participants had heterogeneous professional profiles. While the entire sample filled in the ERQ-adapted, a subsample of 142 participants (93 females; 43 males; 6 missing) also responded to the Big Five Personality Inventory (BFI-Fr; Plaisant, Courtois, Réveillère, Mendelsohn, & John, 2010). Participants of the subsample were aged between 20 and 61 (*M* = 39.01, *SD* = 9.35). Participants signed consent forms and filled in the questionnaires which were subsequently given directly to the researchers or sent to the university via pre stamped-addressed envelopes.

### Measures

#### Emotion Regulation Dispositions

The ERQ-adapted was presented to the participants French-items of the original version were available by Christophe et al. (2009) and used in the present study. Two expressive enhancement items were added to the original scale – “When I want to feel more positive emotions, I *increase their expression*” and “In certain situations, I *increase* the expression of what I am feeling (*be it positive or negative*)”. Items for the new scale were selected from a set of questions related to expressive enhancement, and were created specifically for the study. This choice was made and validated by a group of researchers from the University of Luxembourg, who are specialists in the study of emotional regulation and psychometrics. None of the items related to negative expressive enhancement were retained in the final version of the scale. Indeed questions such as "When I want to feel more negative emotions, I *increase their expression*", were not considered relevant for the questionnaire. This conclusion was verified empirically by a pre-study in which all respondents answered in the negative to these type of questions. These items were therefore not retained in the final version used in this study. The ERQ-adapted assesses the self-perceived frequencies or dispositions of individuals to use *reappraisal* (RD; 6 items), *expressive suppression* (SD; 4 items) and *expressive enhancement* (ED; 2 items). Participants were presented with 12 affirmations; towards which they were asked to indicate their agreement or disagreement on a 7-point Likert scale, ranging from 1 “*strongly disagree*” to 7 “*strongly agree*” – e.g. “I control my emotions *by changing the way I think about the situation*” (reappraisal item) or “I control my emotions *by not expressing them*” (expressive suppression item). A high score in each subscale means a high tendency (or self-perceived frequency) to use the corresponding strategy.

#### Personality

Personality dimensions were assessed using a French version of the Big Five Inventory (BFI; Plaisant et al., 2010). This scale comprises 45 items assessing *Extraversion* (8 items), *Agreeableness* (10), *Conscientiousness* (9), *Neuroticism* (8) and *Openness to Experience* (8). Participants were presented with affirmations concerning these five dimensions of personality (e.g., “I am talkative” for extraversion) and indicate their degree of agreement or disagreement on a 5-point Likert-scale ranging from 1 “*strongly disagree*” to 5 “*strongly agree*”. A mean score was computed for each subscale. A high subscale score indicates that the individual rates highly on that personality dimension.

## Results

### Descriptive Statistics, Internal Consistency and Intra-Scale Correlations

The ERQ-adapted presents acceptable internal consistency, both when considering the overall scale (α = .75) as well as its sub-dimensions: reappraisal (α = .82), expressive suppression (α = .75) and expressive enhancement (α = .76) (see Table 1). Further, an item analysis was performed by applying classical test theory and item response theory (Microsoft Excel tools using the EIRT add-in; Valois, Houssemand, Germain, & Belkacem, 2011), and this confirmed each item’s psychometric characteristics. Thus, all items revealed appropriate discrimination indices (item-total correlations between .40 and .69) and logistic curves. The tendency to regulate emotions by reappraising the situation is independent from the tendency to suppress the expression of emotions. However, expressive enhancement is slightly positively related to reappraisal (*r* = .19, *p* < .01) and to expressive suppression (*r* = .14, *p* < .05). This means that individuals who frequently enhance the expression of emotions also have a small tendency to use the other two strategies, and vice-versa.

**Table 1:** Descriptive Statistics, Internal Consistency and Intra-Scale Correlations

Instrument	*N*	Min	Max	*M*	*SD*	α	Intra-scale correlations			
							a	b	c	d
Age	255	17	68	37.71	11.41					
ERQ - adapted	255					.75				
a. Reappraisal		1.17	7.00	4.68	1.10	.82		.07	.19**	
b. Expressive Suppression		1.00	7.00	3.41	1.31	.75			.14*	
c. Expressive Enhancement		1.00	6.50	3.11	1.48	.76				
BFI	142					.73				
a. Extraversion		1.38	5.00	3.42	0.79	.85				
b. Agreeableness		2.60	5.00	3.94	0.50	.75	.09			
c. Conscientiousness		1.22	5.00	3.93	0.58	.82	.19*	.14		
d. Neuroticism		1.13	4.63	2.81	0.75	.83	-.15	-.17*	-.23**	
e. Openness to expressive		1.70	5.00	3.56	0.64	.81	.18*	.08	.08	.03

*Note*. ERQ = Emotion Regulation Questionnaire; BFI = Big Five Personality Inventory.

*p < .05. **p < .01.

### Factor Structure of the Adapted Version

For this adapted version of the ERQ to be valid, it is to be expected that it presents a three-factor structure. This would include the original reappraisal and suppression factors (comprising respectively, reappraisal and suppression items), and a third factor representing expressive enhancement. Firstly, non-graphical solutions to scree tests were computed on data with Psych and Nfactors R-packages. These analyses confirmed an optimal structural solution composed of three factors. Secondly, exploratory factor analysis (EFA) using method minres (minimum residual method adapted for ordinal data) and principal components analysis (PCA) based on polychoric correlations matrix (adapted for ordinal data), with varimax rotation, were conducted to test whether the theoretically expected structure was observed empirically. For these analyses, the R-Packages Psych and GPArotation were used.

The ERQ-adapted revealed empirically the expected factor structure (Table 2). For EFA, Factor 1 comprises the reappraisal items (Items 1, 3, 5, 7, 8, 11) and it explains 23% of the variance. Factor 2 comprises the suppression items (Items 2, 4, 6, 10) explaining 15% of the variance. The addition of the two enhancement items (Items 9 and 12) reflected statistically by the addition of a third factor which explains 11% of the variance. The total percentage of variance explained by the observed three factors is 49%. For PCA, the results are convergent and the three factors explain respectively 27%, 19% and 14% of explained variance of the items.

**Table 2:** Loadings of ERQ-Adapted Items

Item	Factor 1-Reappraisal		Factor 2-Suppression		Factor 3-Enhancement		Communalities	
	EFA^a^	PCA^b^	EFA^a^	PCA^b^	EFA^a^	PCA^b^	EFA^a^	PCAP^b^
% of variance explained	23	27	15	19	11	14		
1. When I want to feel more positive emotion (such as joy or amusement), I change what I’m thinking about.	.59	.68	.05	.06	-.05	-.11	.36	.48
2. I keep my emotions to myself.	.04	.04	.73	.82	-.02	-.06	.54	.68
3. When I want to feel less negative emotion (such as sadness or anger), I change what I’m thinking about.	.66	.73	-.06	-.06	-.09	-.13	.44	.56
4. When I am feeling positive emotions, I am careful not to express them.	-.13	-.17	.53	.65	.22	.28	.34	.53
5. When I’m faced with a stressful situation, I make myself think about it in a way that helps me stay calm.	.54	.62	.09	.09	.08	.10	.30	.41
6. I control my emotions by not expressing them.	.04	.04	.83	.85	-.05	-.07	.69	.73
7. When I want to feel more positive emotions, I change the way I’m thinking about the situation	.77	.80	.03	.03	.11	.11	.61	.66
8. I control my emotions by changing the way I think about the situation I’m in.	.62	.69	.07	.07	.13	.16	.41	.51
9. When I want to feel more positive emotions, I increase their expression	.11	.11	.16	.16	.84	.86	.74	.77
10. When I am feeling negative emotions, I make sure not to express them.	.21	.22	.54	.67	.14	.15	.36	.52
11. When I want to feel less negative emotion, I change the way I’m thinking about the situation.	.76	.80	-.01	-.01	.10	.11	.59	.65
12. In certain situations, I increase the expression of what I am feeling (be it positive or negative).	.05	.04	.03	.00	.69	.87	.48	.77

*Note*. EFA = Exploratory Factor Analysis; PCA = Principal Component Analysis; ERQ = Emotion Regulation Questionnaire.

^a^Rotation Varimax, Extraction Method: Minres. ^b^Rotation Varimax.

Finally, a structural analysis based on Item Response Models (MIRT R-package) confirmed the previous results and the three-factor structure of the ERQ-adapted (Table 3).

**Table 3:** Loadings of ERQ-Adapted Items (Structural Analysis Based on Item Response Models)

Item	Factor 1-Reappraisal	Factor 2-Suppression	Factor 3-Enhancement	Communalities
% of variance explained	29	19	13	
1. When I want to feel more positive emotion (such as joy or amusement), I change what I’m thinking about.	**.66**	.05	-.01	.45
2. I keep my emotions to myself.	.02	**.80**	.06	.63
3. When I want to feel less negative emotion (such as sadness or anger), I change what I’m thinking about.	**.73**	-.02	-.06	.54
4. When I am feeling positive emotions, I am careful not to express them.	-.13	**.57**	.19	.38
5. When I’m faced with a stressful situation, I make myself think about it in a way that helps me stay calm.	**.62**	-.10	.09	.40
6. I control my emotions by not expressing them.	.06	**.86**	-.01	.73
7. When I want to feel more positive emotions, I change the way I’m thinking about the situation.	**.84**	.03	.11	.73
8. I control my emotions by changing the way I think about the situation I’m in.	**.73**	.09	.10	.55
9. When I want to feel more positive emotions, I increase their expression.	.11	.08	**.98**	.97
10. When I am feeling negative emotions, I make sure not to express them.	.19	**.57**	.19	.40
11. When I want to feel less negative emotion, I change the way I’m thinking about the situation.	**.84**	.01	.10	.71
12. In certain situations, I increase the expression of what I am feeling (be it positive or negative).	-.01	.01	**.69**	.48

*Note*. ERQ = Emotion Regulation Questionnaire. Bold values represent highest loading of each ERQ-adapted item.

### Links Between Emotion Regulation Dispositions and Sex

One-way ANOVA analysis was conducted. Results show, as expected, that men and women do not present significant differences on the self-perceived use of reappraisal (*M*_men_ = 4.73, *SD*_men_ = 1.17; *M*_women_ = 4.64, *SD*_women_ = 1.06; *F*(1, 247) = 0.41, *p* > .05), and men are significantly more disposed to suppress than women (*M*_men_ = 3.89, *SD*_men_ = 1.28; *M*_women_ = 3.13, *SD*_women_ = 1.27; *F*(1, 247) = 20.72, *p* < .05, ω^2^ = .07, *f* = .29). Regarding the self-perceived use of enhancement as a strategy to regulate emotion, data indicates that, contrary to which might be expected, men are significantly more disposed to enhance than women (*M*_men_ = 3.43, *SD*_men_ = 1.45; *M*_women_ = 2.90, *SD*_women_ = 1.45; *F*(1, 247) = 8.05, *p* < .05, ω^2^ = .03, *f* = .18). The observed significant effects of sex on emotion regulation dispositions are small (for enhancement) and medium (for suppression) (Cohen, 1988).

### Links Between Emotion Regulation Dispositions and Age

An initial correlational analysis revealed that suppression and enhancement dispositions are not significantly related to individuals’ age, but reappraisal disposition presents a small positive correlation with age (*r* = .15, *p* < .05). This indicates that the self-perceived tendency to reappraise increases as the individuals get older. For a more comprehensive interpretation of this result, five groups were created based on the participants’ age, and one-way ANOVA were conducted to investigate significant differences between the groups (Table 4).

**Table 4:** Age Differences in the Self-Perceived Use of Emotion Regulation Strategies

Variable	Age group										*F*(4, 247)	*p*	ω^2^
	Group 1^a^ (17-27)		Group 2^b^ (28-37)		Group 3^c^ (38-47)		Group 4^d^ (48-57)		Group 5^e^ (58-67)				
	*M*	*SD*	*M*	*SD*	*M*	*SD*	*M*	*SD*	*M*	*SD*			
Reappraisal	4.40	1.11	4.54	1.06	4.87	1.05	5.00	0.94	4.43	1.78	3.04	.02	.03
Expressive suppression	3.51	1.49	3.42	1.22	3.34	1.30	3.36	1.28	3.86	1.44	0.39	.82	
Expressive enhancement	3.09	1.57	3.15	1.42	3.07	1.49	3.14	1.46	3.39	1.71	0.10	.98	

^a^*N* = 54. ^b^*N* = 87. ^c^*N* = 49. ^d^*N* = 53. ^e^*N* = 9.

Results show that age does not significantly affect the self-perceived use of suppression, *F*(4, 247) = 0.39, *p* = .818, or enhancement, *F*(4, 247) = 0.10, *p* = .982. Nonetheless, the difference in the self-perceived use of reappraisal between the five groups of age was revealed to be statistically significant although small in size, *F*(4, 247) = 3.04, *p* <.05, ω^2^ =.03, *f* = .22. Post-hoc tests using Hochberg’s GT2 (to account for different sample sizes) revealed significant differences between group 1 (*M* = 4.40, *SD* = 1.11) and group 4 (*M* = 5.00, *SD* = 0.94), but no other significant differences were observed (involving Groups 2, 3 and 5). The self-perceived tendency to suppress and enhance appears to be identical regardless of individuals’ age, however. Individuals aged between 48-57 years old report reappraising significantly more often than individuals aged between 17 and 27.

### Links between Emotion Regulation Dispositions and Personality

As reported in Table 5, the tendency to reappraise is not significantly related to any personality trait. The tendency to suppress the expression of emotions is negatively related to extraversion (*r* = -.41, *p* < .01) and the disposition to enhance the expression of emotions is negatively related to conscientiousness (*r* = -.25, *p* < .01). In other words, being introverted is linked with a more frequent use of suppression and being less controlled by conscience/reason. Whereas being spontaneous and permeable to emotions and impulses (low conscientiousness) is linked with a greater use of expressive enhancement.

**Table 5:** Spearman Correlation Between Personality Traits and Self-Perceived Use of Emotion Regulation Strategies

Variable	Extraversion	Agreeableness	Conscientiousness	Neuroticism	Openness
Reappraisal	.10	.10	.08	-.06	.09
Expressive Suppression	-.41**	-.08	-.12	.09	-.13
Expressive Enhancement	.01	-.14	-.25**	.11	.02

**p < .01.

## Discussion – Conclusion

The original ERQ was adapted in order to complement its dimensions with a third dimension addressing expressive enhancement. Two items were developed in French and tested with a francophone sample. The choice of two items was made in order to maintain a balance between reappraisal (6 items), response modulation (6 items – 4 suppression and 2 enhancement), and to avoid redundancy. Further, acknowledging the debates on the necessary number of items per subscale (Bollen, 1989; Marsh, Hau, Balla, & Grayson, 1998), there are methodological arguments suggesting that 2-item and even 1-item dimensions can be justifiable (e.g., Gogol et al., 2014; Nagy, 2002; Wanous, Reichers, & Hudy, 1997). Indeed, the addition of this 2-item dimension modified the original ERQ structure in the expected sense, by creating a new factor comprising the new expressive enhancement items. Further, the results of this study indicate that the addition of these items revealed the expected three-factor structure which explains an important percentage of variance (61%), and has adequate goodness-of-fit indices, as well as good internal consistency overall and by dimension. In other words, three dimensions (reappraisal, expressive suppression and expressive enhancement) were extracted by exploratory factor analysis applying classical test theory and item response theory, with the items’ loadings on theoretical unexpected factors being small. In addition, the items of each dimension present good homogeneity (adequate Cronbach alphas), and the correlation between reappraisal and expressive suppressive is small. This supports the argument that the tendency to use one does not affect the tendency to use the other (Gross & John, 2003). In the case of expressive enhancement and reappraisal, the correlation is small but attains .19 – which suggests that individuals who tend to reappraise have also a low tendency to enhance and vice-versa. This information allows researchers to investigate these two opposite response modulation strategies with some confidence as to the psychometrical properties of this adaptation.

Secondly, individual differences on these strategies and their respective correlates with personality were studied. Regarding gender, data replicates that the tendencies of men and women to reappraise are similar, and men tend to suppress more often than women. As such, men appear to be more predisposed to regulate their emotions by concealing their visible signs. As for expressive enhancement, unexpectedly, our results suggest that men tend to use expressive enhancement significantly more often than women. This result is of interest as it questions our idea that having a higher tendency to display emotions (in this case for women; Buck et al., 1974; Kret & De Gelder, 2012; Kring & Gordon, 1998) would motivate a more frequent use of enhancement. As for the data, even though men tend to display fewer emotions than women, and they regulate their emotions by suppressing their expression more frequently than women (e.g., Gross & John, 2003), they also report a greater use of expressive enhancement. One could interpret this finding as follows: because spontaneously men tend to express less emotion than women (Buck et al., 1974). This strategy might be perceived as being more necessary and useful for them when they want to up-regulate their emotions. In contrast, because emotional expression by women is already generally greater than by men, their use of up-regulation strategies may be perceived as being less necessary and less frequently used.

In terms of age, it was expected that the use of reappraisal would increase, expressive suppression would decrease, and expressive enhancement would not be affected by age. Results confirm the reappraisal and enhancement predictions, however, they indicate that the self-reported use of suppression is not significantly lower for older individuals. Regarding reappraisal, as expected, older individuals (aged from 48-57 years old) report using this cognitive change strategy significantly more often than younger individuals (aged from 17-27 years old). This suggests that, between beginning and more-advanced period of life experience, people appear to develop more cognitive-based methods of dealing with their emotions, experience the advantages of being able to regulate emotions in the desired direction, and thereby increase the use of these strategies. It is particularly noteworthy that an age-related increase in the use of reappraisal does not imply an age-related reduction in the use of strategies focused on modulating the visible signs of an installed emotion (particularly suppression). As expected, no age effects were observed for expressive enhancement. The self-perceived frequency at which individuals up-regulate their emotions by amplifying their visible signs is the same regardless of age group. This suggests that life experience does not impact the perceived utility of this strategy, and nor whether it leads to individuals significantly increasing or decreasing its use. Contrary to our hypothesis, the use of expressive suppression does not significantly decrease with age, but remains identical for different age groups. If the self-perceived tendency to suppress does not decrease as individuals grow older, this could be explained by an assessment of the utility of this strategy that does not change over a life. This evidence points to these types of regulation (expressive suppression and enhancement) appearing to be perceived by individuals as useful (and used) at different stages of life. Further, both in terms of suppression and enhancement, instead of an absolutist maladaptive categorization (particularly in the case of suppression), research has also shown that these strategies are also linked with positive outcomes (Bonanno & Keltner, 1997; Côté et al., 2010) which can contribute to explaining why a decrease in its use was not observed.

Finally, this study analyzed the correlations between these three emotion regulation strategies and personality. The hypotheses tested were: that a high self-reported use of reappraisal would be negatively linked to neuroticism; suppression would correlate negatively with extraversion; and enhancement would correlate positively with openness and negatively with conscientiousness. Like the original ERQ (Gross & John, 2003), data on this adapted version indicates that the self-perceived use of expressive suppression has a moderate negative correlation with extraversion. Introverted people express themselves less in social situations and fear potential rejection, which expectably links to a greater tendency to regulate their emotions by concealing their expression, as it protects them from being exposed to these risks. The results for reappraisal did not confirm the expected negative link to neuroticism. Neurotic individuals are characterized by greater emotional instability and a tendency to feel overwhelmed by negative emotions (Gross & John, 1998). These traits have been shown previously to be linked to a lower disposition to regulate emotions by reappraising situations (Gross & John, 2003). That this was not observed might be indicative that emotionally unstable individuals may use this strategy. However, although they may deploy it, as emotionally stable individuals do, they may not necessarily be capable of doing so effectively. On the other hand, it may reflect potential social desirability bias associated with self-reported measures.

Our exploratory hypothesis tested whether expressive enhancement would be negatively correlated with conscientiousness, and positively correlated with extraversion. Results confirms that highly conscientious individuals report less use of expressive enhancement. This is because they present more controlled behavior, which would be preferably governed by conscience/reason rather than emotion. This contrasts with low conscientiousness, more impulsive individuals. To enhance the expression of emotion might not be a preferable strategy when regulating one’s emotions. Contrary to expectations, extraverts do not seem to use expressive enhancement more often than introverts. The line of thought guiding this hypothesis was based on the fact that extraverts, in contrast to introverts, use less suppression to regulate their own emotions (Cabello et al., 2012; Gross & John, 2003; Ioannidis & Siegling, 2015), and express more openly both positive and negative emotions (Anderson, John, Keltner, & Kring, 2001). However, this result suggests a different interpretation. The ERQ (and the adapted version proposed in this study) assesses regulation of emotions and not their expression. If extraverts spontaneously express more emotions, it can also be the case that when questioned about whether they up-regulate their emotions by enhancing their expression, they might perceive this strategy as not being particularly necessary, and thereby not a strategy they use (in comparison with introverts).

To sum up, an adapted version of the ERQ is pertinent. It complements the original reappraisal and expressive suppression dimensions with the measurement of expressive enhancement. This study sought to investigate whether the modification of the scale would affect its psychometrical properties. The results indicate that the ERQ-adapted has appropriate psychometrical properties, including the expected three-factor structure, which adjusts properly to the data. The gender and age differences observed are partially in line with previous research about the ERQ. Men tend to use expressive suppression significantly more than women, and older individuals tend to reappraise more than younger people (Gross & John, 2003). However different age-groups have similar tendencies to suppress. As for the new expressive enhancement suppression, men report enhancing the expression of emotions more often than women, and the use of this strategy does not vary across age group. Finally, the relationship between personality and these emotion regulation strategies were studied. Results replicated to suggest that introverts are greater “suppressors” than extraverts (Gross & John, 2003), but without supporting the negative link between reappraisal and neuroticism. Finally, regarding expressive enhancement, our results suggest that up-regulating emotions by amplifying expressions is reported less frequently by individuals with high levels of conscientiousness than those with low-conscientiousness traits.

### Limitations

As with all research, this study has a number of limitations and thus should be regarded as a first analysis of the proposed adaptation of the ERQ. Even though this initial data gives evidence of the reliability and construct validity of the scale, further more detailed research will have to be conducted to assure its psychometric qualities and its validation.

In the present study, the number of persons in the sample was relatively small, with only half answering the questions relating to emotional regulation as well as the personality dimensions. In addition, the respondents were people working in Luxembourg, who thus live in a particular cultural, economic, social and linguistic context. Thus the size and characteristics of the sample do not allow for a complete generalization of the results. Further empirical research, with larger and more heterogeneous samples, will be required to verify the robustness and replicability of these findings.

The current analyses were exploratory and will have to be confirmed by analyses which will seek to verify the latent three-factor structure of ERQ. Further more detailed research testing on convergent and divergent links, as well as criteria validity, is still necessary. In this way, the structural and construct validity of the scale can be verified by Structural Equation Modeling (SEM mixing Confirmatory Factor Analyses and Path Analysis). This would integrate the measures of the psychological dimensions of the present study along with others. Thus this allows the highlighting of a heuristic model of emotional regulation processes and its individual sources of variation.

Finally, there is further area of particular relevance for future research. Since the processes of emotional regulation seems to vary according to life experience (even if these changes are not totally monotonic), it would be interesting to complement cross-sectional data with longitudinal data. This is the only method by which to draw conclusions on an intra-individual variation of emotional regulation processes during different lifespans. This would allow more straightforward conclusions to be drawn about individuals’ development in the use of these emotion regulation strategies.
